# Integration of CT urography improves diagnostic confidence of ^68^Ga-PSMA-11 PET/CT in prostate cancer patients

**DOI:** 10.1186/s40644-017-0132-6

**Published:** 2017-12-20

**Authors:** Leon Will, Frederik L. Giesel, Martin T. Freitag, Anne K. Berger, Walter Mier, Klaus Kopka, Stefan A. Koerber, Hendrik Rathke, Christophe Kremer, Clemens Kratochwil, Hans-Ulrich Kauczor, Uwe Haberkorn, Tim F. Weber

**Affiliations:** 1 Department of Nuclear Medicine, Heidelberg University Hospital, Im Neuenheimer Feld 400, 69120 Heidelberg, Germany; 2 Cooperation Unit Nuclear Medicine, German Cancer Research Center (dkfz), Im Neuenheimer Feld 280, 69120 Heidelberg, Germany; 3 Division of Radiology, German Cancer Research Center (dkfz), Im Neuenheimer Feld 280, 69120 Heidelberg, Germany; 4 Department of Medical Oncology, National Center for Tumor Diseases, Heidelberg University Hospital, Im Neuenheimer Feld 460, 69120 Heidelberg, Germany; 5 Division of Radiopharmaceutical Chemistry, German Cancer Research Center (dkfz), Im Neuenheimer Feld 280, 69120 Heidelberg, Germany; 6 Department of Radiation Oncology, Heidelberg University Hospital, Im Neuenheimer Feld 400, 69120 Heidelberg, Germany; 7 Department of Diagnostic and Interventional Radiology, Heidelberg University Hospital, Im Neuenheimer Feld 110, 69120 Heidelberg, Germany

**Keywords:** PSMA, Pet/Ct, CT urography, Prostate cancer, Renal excretion, Staging

## Abstract

**Background:**

To prove the feasibility of integrating CT urography (CTU) into ^68^Ga-PSMA-11 PET/CT and to analyze the impact of CTU on assigning focal tracer accumulation in the ureteric space to either ureteric excretion or metastatic disease concerning topographic attribution and diagnostic confidence.

**Methods:**

Ten prostate cancer patients who underwent ^68^Ga-PSMA-11 PET/CT including CTU because of biochemical relapse or known metastatic disease were retrospectively analyzed. CTU consisted of an excretory phase 10 min after injection of 80 mL iodinated contrast material. Ureter opacification at CTU was evaluated using the following score: 0, 0% opacification; 1, < 50%; 2, 50–99%; 3, 100%. Topographic attribution and confidence of topographic attribution of focal tracer accumulation in the ureteric space were separately assessed for ^68^Ga-PSMA-11 PET/CT without and with CTU. Diagnostic confidence was evaluated using the following score: 0, < 25% confidence; 1, 26–50%; 2, 51–75%; 3, 76–100%.

**Results:**

At CTU, mean ureter opacification score was 2.6 ± 0.7. At ^68^Ga-PSMA-11 PET/CT without CTU, mean confidence of topographic attribution of focal tracer accumulation was 2.5 ± 0.7 in total and 2.6 ± 0.7 for metastatic disease. At ^68^Ga-PSMA-11 PET/CT with CTU, mean confidence of topographic attribution of focal areas of tracer accumulation was significantly higher with 2.9 ± 0.2 in total and 2.7 ± 0.9 for metastatic disease (*p* < 0.001). In 4 of 34 findings (12%) attribution to either ureteric excretion or metastatic disease was discrepant between ^68^Ga-PSMA-11 PET/CT without and with CTU (n.s).

**Conclusions:**

Integration of CTU into ^68^Ga-PSMA-11 PET/CT is feasible and increases diagnostic confidence of assigning focal areas of tracer accumulation in the ureteric space to either metastatic disease or ureteric excretion.

## Background

Prostate Cancer is the most common malignancy in men and the third most frequent cause of cancer-associated death [1, 2]. With the introduction of prostate-specific antigen (PSA) screening, many patients are diagnosed with localized disease, but still a subset of patients develops high-risk or metastatic disease. After radical treatment of localized disease, biochemical relapse according to PSA elevation occurs in approximately 30% of prostate cancer patients [3].

Accurate diagnosis of prostate cancer relapse, identification of involved anatomical sites, and assessment of tumor load are crucial for treatment stratification and metastases-directed therapies such as salvage lymph node dissection. Early detection of prostate cancer relapse is, however, a major challenge for conventional imaging methods including computed tomography (CT) and magnetic resonance imaging (MRI) [4]. Functional imaging techniques targeting the prostate-specific membrane antigen (PSMA) using positron emission tomography (PET) have shown great potential for detection of prostate cancer and its metastases [5]. A key step in PET imaging with PSMA ligands was the development of ^68^Ga-labelled PSMA (^68^Ga-PSMA-11) [6]. ^68^Ga-PSMA-11 binds to the extracellular part of the PSMA receptor and is then internalized into the prostate cancer cell.

^68^Ga-PSMA-11 is eliminated via the renal pathway, so that detection of disease with PET/CT using ^68^Ga-PSMA-11 may be limited by topographic proximity of tumor sites with the urinary tract or by superimposition of tumor sites and ureteric tracer accumulation [7]. Furthermore, the low-dose CT scan of a conventional ^68^Ga-PSMA-11 PET/CT may be limited in regards of anatomical landmarking and, thus, may have limited diagnostic confidence to correctly classify a focal tracer accumulation as tumor uptake or ureteric tracer excretion. Hence, improving visualization of the upper urinary tract using CT urography (CTU) as the first choice imaging technique to depict the urinary tract may have added value for ^68^Ga-PSMA-11 PET/CT if conducted after the regular PET/CT scan.

The aim of this retrospective study was to evaluate the integration of CTU into ^68^Ga-PSMA-11 PET/CT. We hypothesized that availability of a CTU scan improves diagnostic confidence of attributing focal areas of tracer accumulation in the ureteric space to either ureteric tracer excretion or tumor uptake at ^68^Ga-PSMA-11 PET/CT.

## Methods

### Study design and study population

This retrospective single-center exploratory study was approved by the local institutional review board (S-321/2012) and conducted in agreement with the Declaration of Helsinki and its later amendments. In 10 consecutive patients, in which ^68^Ga-PSMA-11 PET/CT was performed for standard clinical indications, ^68^Ga-PSMA-11 PET/CT was supplemented by CTU at the discretion of the attending nuclear medicine physician. Clinical data was extracted from the electronical medical records.

### Imaging

All patients underwent imaging on a Biograph mCT Flow scanner (Siemens, Erlangen, Germany). FlowMotion was used to acquire PET in 3D mode (matrix 200 × 200). Correction for randoms, scatter and decay was performed for the emission data. Images were reconstructed with an ordered subset expectation maximization (OSEM) algorithm with two iterations/21 subsets and Gauss-filtered to a transaxial resolution of 5 mm at full-width at half-maximum (FWHM). Attenuation was corrected using unenhanced low-dose CT reconstructed to a slice thickness of 5 mm with an increment of 3–4 mm.

CTU was performed during the same ^68^Ga-PSMA-11 PET/CT session on the same scanner. All patients received a bolus of 80 mL intravenous iodine-based contrast agent (iopromide, 300 mg per mL, Ultravist, Bayer, Leverkusen, Germany) via injection. The excretory phase of CTU was finally performed 600 s after injection (slice thickness, 2.0 mm; reconstruction increment, 1.6 mm; tube voltage, 120 kVp; reference tube current time product, 190 mAs). Additional means to intensify ureteric opacification at CTU, such as furosemide injection, were not undertaken.

### Image analysis

Two independent analyses of image data sets were performed: First, contrast opacification of the lumen of the ureters at CTU was assessed to investigate the quality of CTU. Second, the confidence of attributing a PET-positive finding in the ureteric space to metastatic disease or ureteric tracer excretion was determined.

In order to assess the contrast enhancement of the ureters two blinded board-certified readers with more than 10 years of experience in abdominal radiology each (FLG, TFW) reviewed CTU images independently on an imaging workstation (Centricity, GE Healthcare, Chalfont St. Giles, Great Britain). The ureters were divided into a proximal segment above the iliac vessels and a distal segment below the iliac vessels. The readers were advised to assign an opacification score to each section according to the CTU analyses published by other groups before [8]. The opacification scores were the following: 0, 0% opacification; 1, < 50% opacification; 2, 50–99% opacification; 3, 100% opacification.

In order to assess the confidence of attributing a PET-positive finding in the ureteric space to tumor uptake or ureteric tracer excretion a two-step analysis was performed by one reader (TFW). First, fused unenhanced ^68^Ga-PSMA-11 PET/CT images were reviewed without consideration of the CTU scan (confidence read 1). Focal areas of tracer accumulation in the ureteric space were identified and attributed either to tumor uptake or ureteric tracer excretion. Diagnostic confidence of assignment was recorded using the following confidence score: 0, < 25% confidence; 1, 26–50% confidence; 2, 51–75% confidence; 3, 76–100% confidence. Second, four weeks after confidence read 1 the examinations were read again including the CTU scan (confidence read 2). PET-positive findings identified at confidence read 1 were anatomically correlated to the CTU scan. Again, diagnostic confidence of attributing focal areas of tracer accumulation in the ureteric space to either tumor uptake or ureteric tracer excretion was scored for each finding.

### Statistics

Interreader agreement of the assessment of ureter opacification was calculated by using the intraclass coefficient (ICC) employing a two-way consistency model indicating that the absolute differences were neglected. The average-measures ICC is given with 95% confidence interval. 0.00 to 0.20 indicates slight agreement, 0.21 to 0.40 fair agreement, 0.41 to 0.60 moderate agreement, 0.61 to 0.80 substantial agreement, and 0.81 to 1.00 almost perfect or perfect agreement. Differences in anatomical attribution of PET-positive findings and differences in diagnostic confidence of anatomical attribution were analyzed using Wilcoxon signed-rank tests for paired samples.

## Results

### Patients

The study group consisted of 10 men with a median age of 70 years (range, 63–81). Indication for ^68^Ga-PSMA-11 PET/CT was biochemical relapse after radical prostatectomy in 8 patients, staging prior to treatment in 1 patient, and re-staging during hormonal therapy in 1 patient, respectively. For 5 patients, long-term oncological follow-up data was available. Of these patients, 4 had confirmed advanced/metastatic disease diagnosed at ^68^Ga-PSMA-11 PET/CT, and tumor-specific therapy was initiated. 1 patient had no morphological sign of disease recurrence despite PSA progression but developed recurrent tumor later on during the course of disease. For the other 5 patients, PSMA CT revealed recurrent advanced diseases in 3 patients, and in 2 cases no tumor activity was detected. Clinical characteristics are summarized in Table 1.

**Table 1:** Clinical characteristics of study patients

Patient number	Age	Extent of disease at ^68^Ga-PSMA-11 PET/CT	Initiation of tumor specific therapy
1	72	Advanced/metastatic	Yes
2	63	Advanced/metastatic	Unknown
3	81	Advanced/metastatic	Unknown
4	67	Advanced/metastatic	Unknown
5	63	No morphological recurrence	Not available
6	71	Advanced/metastatic	Yes
7	71	Local tumor	Yes
8	64	Advanced/metastatic	Yes
9	70	No morphological recurrence	Not available
10	53	No morphological recurrence	Not available

### Opacification score

In total, 40 ureter segments (10 patients, 4 segments per patient) have been evaluated. Ureter opacification was scored by reader 1 with a mean score of 2.5 ± 0.6 and by reader 2 with a mean score of 2.7 ± 0.7. The total opacification score averaged over both readers was 2.6 ± 0.7. Both readers demonstrated near perfect agreement for all ratings taken together (ICC, 0.86 (0.79–0.91)). Table 2 shows the mean score for each segment by each reader and the interreader agreement per segment.

**Table 2:** Ureter opacification scoring and interreader agreement

Segment	ICC (95% CI)	Reader 1	Reader 2
Left proximal ureter	0.96 (0.85–0.99)	2.5 ± 0.9	2.4 ± 0.8
Right proximal ureter	1.00	2.7 ± 0.7	2.7 ± 0.7
Left distal ureter	0.87 (0.49–0.97)	2.0 ± 0.7	2.1 ± 1.0
Right distal ureter	0.82 (0.29–0.96)	2.2 ± 0.4	2.3 ± 0.8
All ratings combined	0.86 (0.79–0.91)	2.5 ± 0.6	2.7 ± 0.7

Reader 1 rated opacification of 34 of 40 segments (85%) with a score of at least 2. Reader 2 rated opacification of 33 of 40 segments (82.5%) with a score of at least 2. The quantitative distribution of ureteric opacification scores is given in Table 3.

**Table 3:** Distribution of ureteric opacification scores

Opacification score	Reader 1 (% of segments)	Reader 2 (% of segments)
3	20 (50)	23 (58)
2	14 (35)	10 (25)
1	6 (15)	6 (15)
0	0 (0)	1 (3)

### Diagnostic confidence

In confidence read 1, considering fused images of the unenhanced PET/CT alone, 34 focal areas of tracer accumulation in the ureteric space were identified. Of these, 14 were attributed to tumor uptake. Mean confidence of attribution in read 1 was 2.5 ± 0.7 in total and 2.6 ± 0.7 for tumor uptake.

**Fig. 1:**
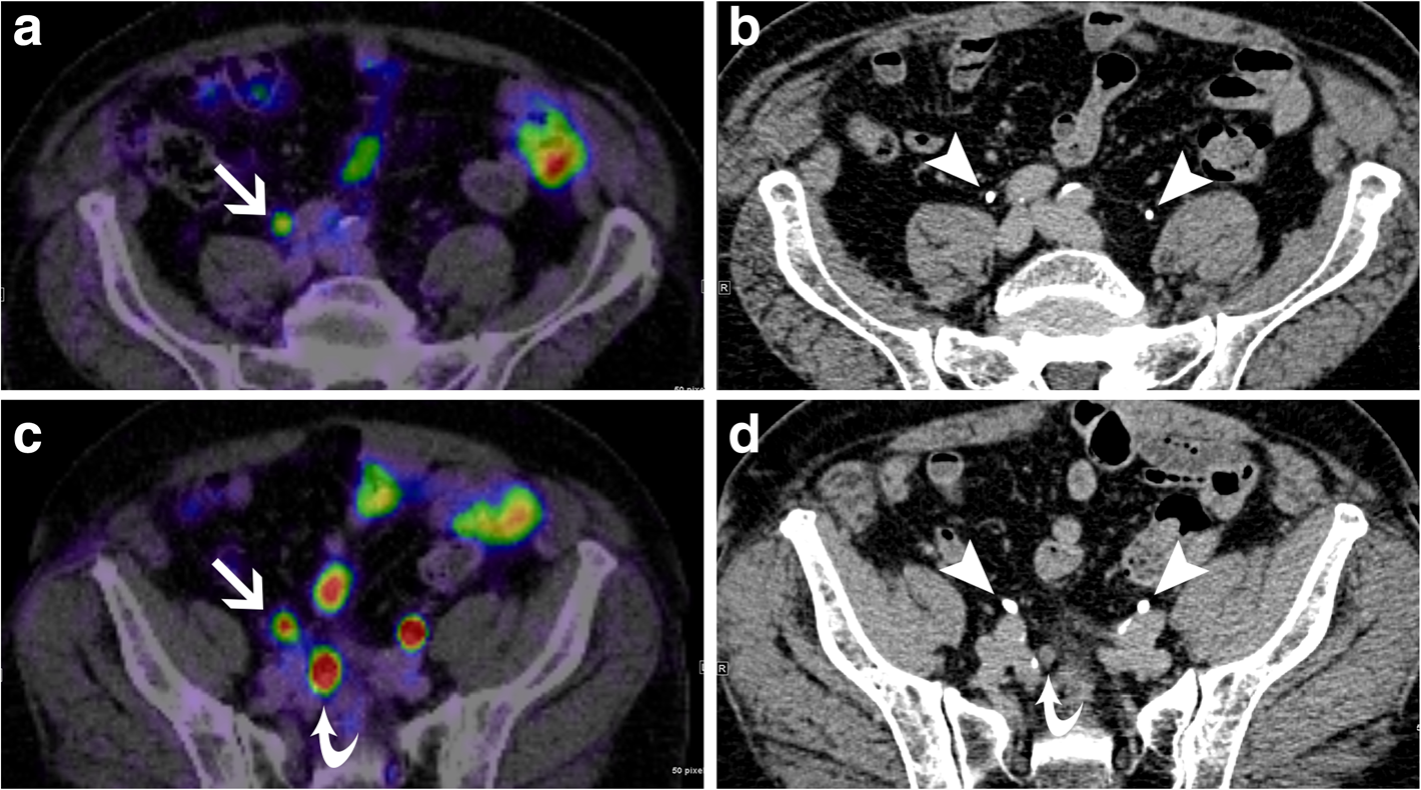
Discrepancies for ^68^Ga-PSMA-11 PET/CT without and with CTU in anatomical assignment of tracer accumulation in a patient with multifocal retroperitoneal nodal relapse. **a** shows focal tracer accumulation in the right iliac retroperitoneum that was assigned to tumor uptake in ^68^Ga-PSMA-11 PET/CT alone (arrow in **a**, confidence score 1) and to right ureteric excretion in ^68^Ga-PSMA-11 PET/CT with CTU (arrow heads in **b** refer to both ureters, confidence score 3). **c** again shows focal tracer accumulation in the right iliac retroperitoneum that was assigned to tumor uptake in ^68^Ga-PSMA-11 PET/CT alone (arrow in **c**, confidence score 1) and to right ureteric excretion in ^68^Ga-PSMA-11 PET/CT with CTU (arrow heads in **b** refer to both ureters, confidence score 3). There is another focal tracer accumulation that was assigned to metastatic disease in ^68^Ga-PSMA-11 PET/CT without and with CTU (curved arrow in **c** and **d**, both confidence score 3)

**Fig. 2:**
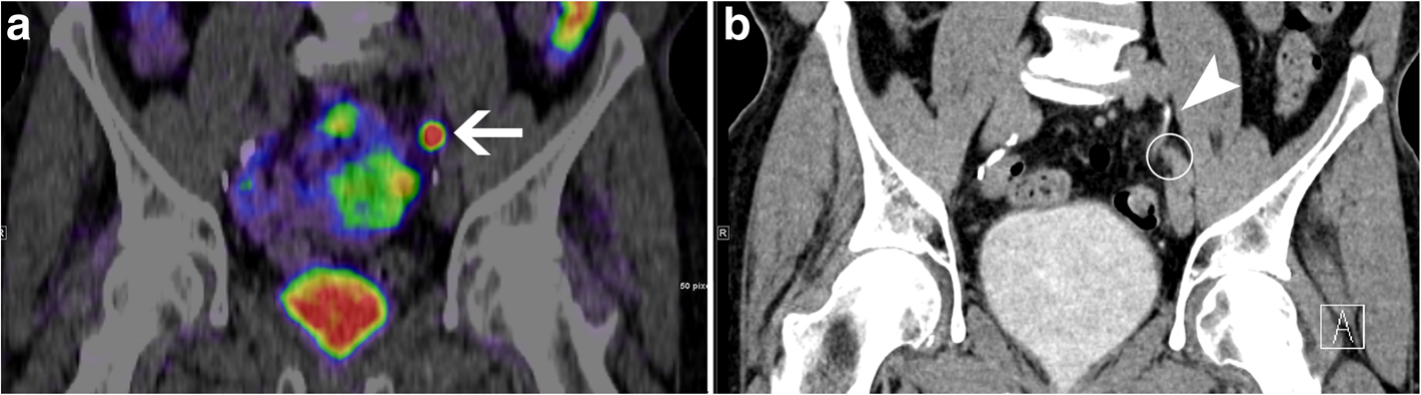
Discrepancies for ^68^Ga-PSMA-11 PET/CT without and with CTU in anatomical assignment of tracer accumulation in a patient with unifocal retroperitoneal nodal relapse according to ^68^Ga-PSMA-11 PET/CT with CTU. **a** shows a focal tracer accumulation in the left retroperitoneum at the the level of the ureteric crossing of the iliac vessels with anatomical assignment to the left ureter with confidence score 1. Using ^68^Ga-PSMA-11 PET/CT with CTU, the focus was assigned to a small periureteric soft tissue mass (circle in **b**) between the left ureter (arrow head in **b**) and the left iliac vessels with confidence score 3

In confidence read 2, considering fused images of the unenhanced PET/CT and CTU together, attribution of focal areas of tracer accumulation to tumor uptake or ureteric excretion was discrepant from confidence read 1 in 4 of 34 findings (12%) or 2 of 10 patients (20%), respectively. Three of these discrepancies occurred in one patient and consisted of attribution of tracer accumulation to ureteric excretion rather than to tumor uptake (Fig. 1). In this patient, other focal areas of tracer accumulation attributed to tumor uptake in both reads were present as well. The remaining discrepancy consisted of attribution of tracer accumulation to tumor uptake rather than to ureteric excretion in a different patient (Fig. 2). In this patient, other focal areas of tracer accumulation attributed to tumor uptake were not present. The confidence score differed from read 1 in 15 findings (44%). In 14 of these 15 findings the confidence score in read 2 was at least 1 grade higher than in read 1. Mean confidence of attribution in read 2 was 2.9 ± 0.2 in total and 2.7 ± 0.9 for tumor uptake.

**Fig. 3:**
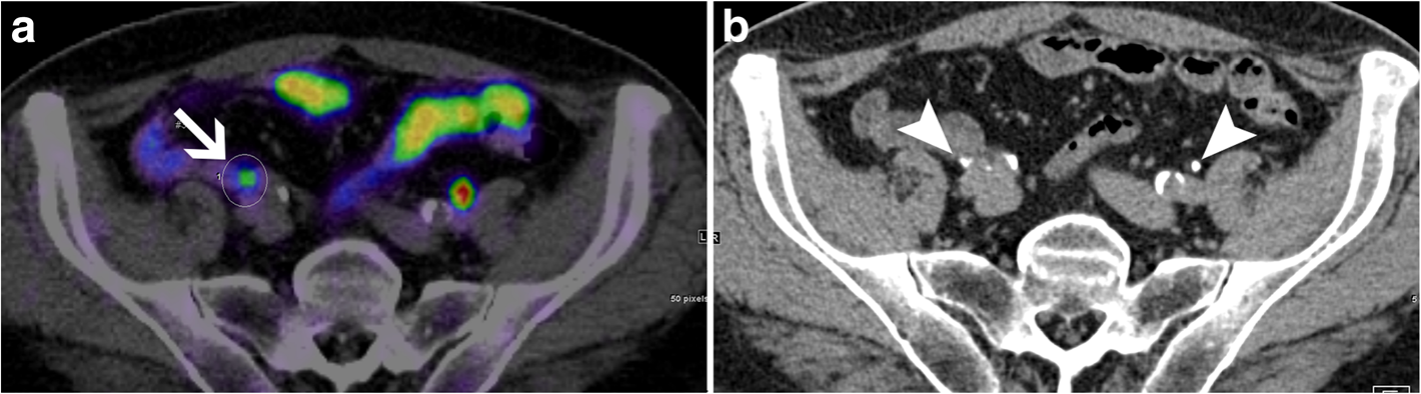
Increase of diagnostic confidence by integrating CTU into ^68^Ga-PSMA-11 PET/CT with CTU. **a** shows a focal tracer accumulation in the right iliac retroperitoneum (arrow in **a**) with anatomical assignment to the right ureter with confidence score 1. Anatomical assignment is hampered by proximity of the focus to small intestine and iliac crossing of the right ureter. Using ^68^Ga-PSMA-11 PET/CT with CTU and delineation of both ureters (arrow heads in **b**), the focus was assigned to right ureteric excretion with confidence score 3

Anatomic attribution of focal areas of tracer accumulation to either tumor uptake or ureteric excretion did not differ significantly between confidence read 1 and 2 in total (*p* = 0.375). The confidence of attribution was significantly higher for the combined read of PET/CT and CTU than for PET/CT alone (*p* < 0.001) (Fig. 3).

### CTU radiation exposure

The mean CT volume dose index (CTDI_vol_) and dose length product (DLP) of CTU were 15 ± 6 mGy and 694 ± 334 mGycm.

## Discussion

The present analysis demonstrates that integration of CTU into ^68^Ga-PSMA-11 PET/CT imaging of prostate cancer patients is feasible and increases diagnostic confidence of attribution of focal areas of tracer accumulation in the ureteric space to either tumor uptake or ureteric excretion. In a considerable amount of focal areas of tracer accumulation integration of CTU changed the assessment of anatomic allocation (12%) and, thus, may impact on cancer patient management in select cases.

Nowadays, ^68^Ga-PSMA-11 PET/CT plays an important role in management of prostate cancer patients [9]. Especially in the event of biochemical relapse, early detection of recurrent disease is of major importance to identify individuals that are eligible for curative treatment options including salvage lymphadenectomy. In a study of 319 patients with biochemical failure after curatively intended therapy more than 80% of patients had at least one positive lesion at ^68^Ga-PSMA-11 PET/CT [10]. The probability of a positive ^68^Ga-PSMA-11 PET/CT increases with the blood level of PSA: For PSA levels between 0.5 and 1.0 ng/mL it is almost 60% and rises rapidly with increasing PSA levels [10, 11]. In a recent meta-analysis, overall sensitivity and specificity were about 86% for per-patient analysis and 80% and 97% for per-lesion analysis, respectively [11].

A ligand-specific challenge is evaluation of tumor foci in close proximity to ureter and bladder because of urinary excretion of ^68^Ga-PSMA-11 [7, 12]. A focal tracer accumulation in the retroperitoneum and pelvis can be misinterpreted because the low-dose scan typically included in standard PET/CT imaging may not offer sufficient anatomical detail to correctly identify the underlying cause of accumulation, especially for the inexperienced reader. Thus, urinary excretion of ^68^Ga-PSMA-11 may influence data on false positive and false negative lymph node assessment at ^68^Ga-PSMA-11 PET/CT [13–15]. As the problem of identification of local recurrences in the prostate bed, that may be obscured by tracer activity within the bladder, was considered not to be addressed by CTU integration, the ability of detecting local recurrences beneath the ureter orifices was not subject of this study.

Although uncertainty of anatomical attribution of focal areas of tracer accumulation may be present only in a minority of cases, the impact of over- or understaging disease on patient management may be tremendous and may justify integration of an additional CTU scan at least in select cases with equivocal findings at standard ^68^Ga-PSMA-11 PET/CT. Due to the small size of our study cohort and lack of long-term clinical data in half of our patients, impact of CTU integration on specific oncological treatment decisions and patient outcome cannot be derived from the results.

The standard CTU protocol consists of unenhanced, nephrographic phase, and excretory phase imaging. Common general indications for CTU include hematuria, staging and follow-up of urothelial cancer, urinary tract obstruction, and anomalies of urinary tract anatomy [16]. It does not only allow detailed assessment of the urinary tract but also facilitates evaluation of adjacent abdominal and pelvic structures. The urography protocol used in this study was a single-bolus technique with administration of 80 ml of contrast material and excretory phase imaging 10 min after injection. Although the frequently recommended amount of contrast material for CTU is 100 to 150 ml, opacification of the urinary tract was sufficient in our study even in distal ureter segments [17]. As anatomic delineation of the ureter and not evaluation of the upper urinary tract for urothelial masses was the intention for CTU, the reduction of the amount of contrast material was justified. For the same reason we did not use oral hydration, intravenous saline infusion, or furosemide injection to intensify ureter distention - techniques that have been suggested to improve assessment of the upper urinary tract [18–20].

Because a standard dose contrast enhanced CT scan during nephrographic or portal venous phase was not performed in our study, it remains unclear if such an acquisition would provide similar results to CTU regarding anatomic attribution of focal tracer accumulations. A nephrographic or portal venous phase was not acquired because sole visceral metastases are rare in prostate cancer patients, the excretory phase is considered to be superior for exact urinary tract depiction, and it would have added even more additional radiation exposure to the diagnostic procedure. Of note, using a split bolus technique for CTU, simultaneous acquisition of a nephrographic and excretory phase has been introduced as an alternative one-stop-shop CTU technique with reduced radiation dose [8, 21]. CTU was done in our study with standard abdominal scanning parameters with 120 kVp and image reconstruction using filtered back projection. Studies have shown that iterative image reconstruction or reduction of tube voltage to 100 kVp may allow significant dose reduction without decline of CTU image quality [22, 23]. Iterative reconstruction is already available on modern PET/CT scanners and may be preferred over filtered back projection for diagnostic contrast enhanced dose reduced body scans. An alternative to CTU could be to increase ureteric clearance of ^68^Ga-PSMA-11 by injection of furosemide prior to the delayed PET scan and adding another unenhanced low-dose scan of the abdomen and pelvis for anatomical correlation. Derlin et al. have shown that forced diuresis with furosemide injection may reduce linear and focal tracer accumulation in standard ^68^Ga-PSMA-11 PET/CT and, thus, may improve image quality without the need of intravenous contrast administration and CTU scanning [24]. However, correct timing of furosemide injection seems to be crucial in this approach as too early furosemide injection may even deteriorate image quality.

Today, several PSMA ligands aside from ^68^Ga-PSMA-11 are available: ^68^Ga-PSMA-I + T, ^68^Ga-PSMA-617 as well as ^18^F–DCFBC, ^18^F–DCPyL, and, most recently, ^18^F–PSMA-1007. All of these PSMA-ligands have similar characteristics in regards of tumor uptake and tracer clearance except for ^18^F–PSMA-1007 [12, 25–28]. This ligand is cleared primarily via the hepatobiliary route and not via the urinary tract and may improve diagnostic confidence particular in these suspicious areas [12].

### Limitations

This retrospective study is limited by small sample size and, thus, can only be considered hypothesis-generating. It cannot be derived from our data how often integration of CTU into ^68^Ga-PSMA-11 PET/CT may actually change oncological patient management and influence long-term clinical outcome. Larger and prospectively generated cohorts are to be studied to fully assess the clinical benefit of our method. As histological correlation of imaging findings was not undertaken, a gold standard to estimate the diagnostic accuracy of ^68^Ga-PSMA-11 PET/CT with and without CTU including rates of false positive and false negative findings is not available. Data on interrater reliability of the improvement of diagnostic confidence are not available.

## Conclusions

Integration of CTU into ^68^ Ga-PSMA-11 PET/CT may help the imaging specialist in anatomical attribution of questionable findings and enhance confidence in interpretation of ^68^Ga-PSMA-11 PET/CT scans in select cases.

## Abbreviations

CT: Computed tomography
CTDIvol: CT volume dose index
CTU: CT urography
DLP: Dose length product
FWHM: Full-width at half-maximum
ICC: Intraclass coefficient
MRI: Magnetic resonance imaging
OSEM: Ordered subset expectation maximization
PET: Positron emission tomography
PSA: Prostate-specific antigen
PSMA: Prostate-specific membrane antigen
